# Barriers accessing care in a healthcare system suffering from under‐investment: Themes from interviews with 51 people living with Alzheimer's disease and their caregivers in Puerto Rico

**DOI:** 10.1002/alz70860_103866

**Published:** 2025-12-23

**Authors:** Alexandra Mendoza‐Graf, Gabriela Castro, Gilberto Ramos Valencia, Mariela Torres‐Cintrón, Marielena Lara, Ivonne Z. Jimenez‐Velazquez, Ilka Rios, Johan Hernandez Santiago, Beverly Weidmer, Justin W. Timbie

**Affiliations:** ^1^ RAND Corporation, Santa Monica, CA, USA; ^2^ University of Puerto Rico, School of Medicine, San Juan, PR, USA; ^3^ RAND Corporation, Alexandria, VA, USA

## Abstract

**Background:**

Puerto Rico's healthcare system suffers from substantial under‐investment due to a decades‐long period of slowing economic growth, devastating natural disasters, inadequate Medicaid funding (due to the territory's block grant financing mechanism), and significantly lower Medicare Advantage (MA) payments relative to plans operating in the U.S. mainland. A rapidly aging population and an exodus of healthcare specialists to the mainland U.S. compounds the problem. These challenges raise concerns about access to care for Puerto Rico's residents with Alzheimer's disease (AD) who require coordinated care from diverse specialists. We conducted interviews with people living with AD and their primary, family caregiver to identify current barriers accessing care.

**Method:**

We conducted semi‐structured interviews with 51 patient/caregiver dyads, recruited from local support groups and advocacy organizations. Interviews were conducted in Spanish from 12/2023 to 11/2024, recorded, and transcribed. A team of four analysts, meeting inter‐rater reliability benchmarks, coded and thematically analyzed transcripts.

**Results:**

Access barriers most commonly identified by patients and caregivers included five structural barriers: (1) shortages of neurologists; (2) frustration with closed provider panels and restrictive MA plan provider networks that required patients to periodically switch neurologists; (3) lack of coverage for adult day center services (due to the lack of a long‐term services and supports (LTSS) benefit in Puerto Rico's Medicaid program) and limited availability of adult day center services on a self‐pay basis or provided free by municipalities without waiting lists; (4) lack of coverage for personal care services (due to the absence of LTSS); and (5) lack of routine screening for health‐related social needs and caregiver needs despite the ability of MA plans to provide non‐medical supplemental benefits. Two burdensome care processes included: (1) hours‐long wait times before being seen during routine appointments during which family members with AD often became distressed; and (2) antiquated, paper‐based referrals requiring retrieval in‐person from clinics that imposed travel burdens on caregivers.

**Conclusion:**

AD patients in Puerto Rico face significant unmet service needs and access challenges, largely due to inadequate investment in its Medicare and Medicaid programs. Aligning Puerto Rico's Medicaid and MA financing methodologies with that of the mainland U.S. could enhance access to care.

